# Two‐Year Outcomes of Regenerative Therapy in an Immature Maxillary Molar With Periapical Abscess: A Case Report

**DOI:** 10.1155/crid/7835569

**Published:** 2026-04-15

**Authors:** Luiz Felipe Rodrigues Siqueira, Thais Caetano de Souza-Guedes, Laura Ferreira Araujo, Paulo Ricardo de Sousa Pereira, Crisnicaw Veríssimo, Patrícia Correia de Siqueira, Juliano Gonçalves Miguel, Orlando Aguirre Guedes, Gustavo Silva Chaves

**Affiliations:** ^1^ Department of Stomatology, School of Dentistry, Federal University of Goiás, Goiânia, Goiás, Brazil, ufg.br; ^2^ Department of Oral Biology, School of Dentistry, Alfredo Nasser University Center, Goiânia, Goiás, Brazil; ^3^ Department of Endodontics, School of Dentistry, Pontifical Catholic University of Goiás, Goiânia, Goiás, Brazil, pucgoias.edu.br

**Keywords:** dental pulp necrosis, maxillary molar, periapical abscess, regenerative endodontics

## Abstract

Trauma or caries in teeth with incomplete root formation can lead to pulp necrosis. Endodontic treatment in such cases requires specific clinical protocols that differ from conventional approaches due to the anatomical and developmental characteristics of immature teeth. This case report describes the clinical management of a maxillary second molar (tooth #17) with incomplete root development and a periapical abscess, treated through pulp revascularization. A 13‐year‐old male patient presented with spontaneous, throbbing pain in the upper right facial region, accompanied by infraorbital swelling. Intraoral examination revealed extensive loss of tooth structure in tooth #17 and the presence of a palatal abscess. Cold pulp testing was negative, while percussion testing was positive, supporting the diagnosis of periapical abscess. Due to the extensive tooth structure, a crown lengthening procedure was performed. Endodontic access was achieved under rubber dam isolation, and three root canals were identified and carefully emptied. Irrigation was performed with 2.5% sodium hypochlorite 4 mm short from the working length, followed by 3 min of EDTA application. Ultracal was used as intracanal medicament for 30 days. At the second appointment, clot induction was achieved with a #20 file, and the cervical third was sealed with Biodentine, followed by composite resin restoration. After a 2‐year clinical, radiographic, and tomographic follow‐up, the tooth remained asymptomatic, with radiographic evidence of continued root maturation and apical closure, suggesting a favorable outcome of the regenerative protocol.

## 1. Introduction

Dental caries is highly prevalent and often affects recently erupted permanent teeth, such as maxillary molars, which are more prone to pulp involvement due to their large pulp chambers and lower enamel mineralization [1]. When pulp necrosis occurs during root development, treatment planning must consider the degree of root maturation and pulp vitality to define the best approach, apexogenesis, apexification, or regenerative procedures [1, 2].

Immature teeth present anatomical challenges, such as wide canals, open apices, and absence of natural constriction, which hinder work length determination and obturation [2, 3]. Among the complications of pulp necrosis, periapical abscesses are marked by localized pain, swelling, and purulent exudate accumulation. Histologically, these lesions are characterized by intense neutrophilic infiltration and tissue destruction [4].

Regenerative endodontic procedures (REPs) have been proposed as a biologically based treatment strategy capable of promoting root development and apical closure in immature teeth with necrotic pulp [1, 2, 5–7]. Unlike traditional apexification techniques, which mainly induce the formation of a calcified apical barrier without further root maturation, REPs aim to restore a functional pulp‐like tissue and allow continued root development [7, 8]. Although several reports have demonstrated successful outcomes using this approach in single‐rooted teeth [7, 9, 10], cases involving immature maxillary molars remain relatively scarce [6]. Therefore, the present report describes the regenerative endodontic management of an immature maxillary molar with a periapical abscess and a 2‐year follow‐up.

## 2. Case Report

A 13‐year‐old boy, accompanied by his legal guardian, presented to the dental emergency service reporting spontaneous, intense, localized, and pulsatile pain on the right side of the maxilla, associated with infraorbital swelling. The boy’s medical history was noncontributory.

**Figure 1:**
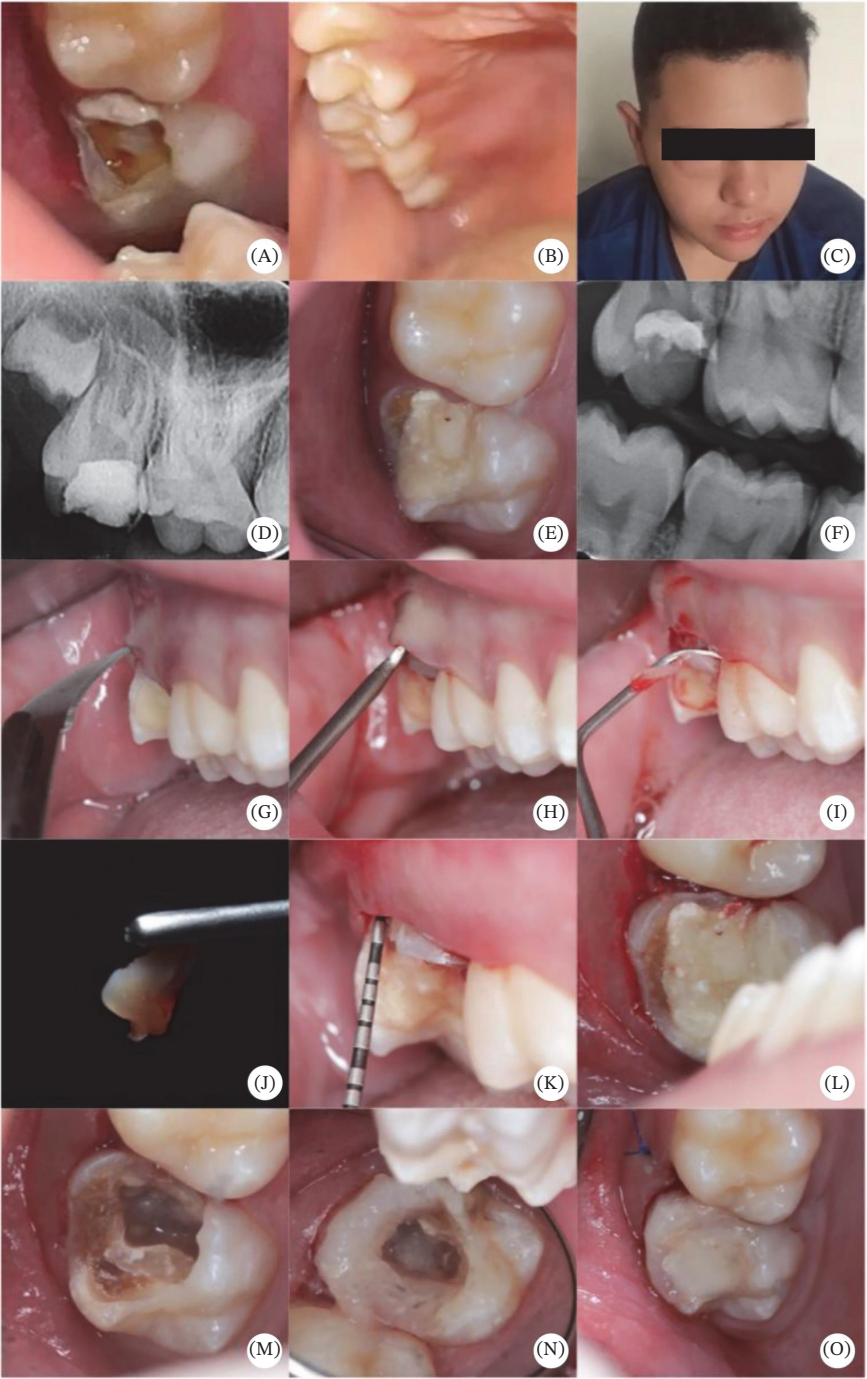
Emergency dental care and clinical crown lengthening with gingival margin elevation. (A–C) Initial clinical presentation, including extensive coronal destruction of tooth #17 and extraoral swelling. (D–F) Radiographic and intraoral assessment confirming incomplete root development and periapical involvement. (G–J) Periodontal surgical procedures for crown lengthening, including internal bevel incision, flap elevation, and removal of the cervical tissue collar. (K–O) Postoperative evaluation showing re‐establishment of the gingival margin, stabilization of the surgical site, and final aspect after suturing.

Clinical examination revealed extensive coronal destruction of tooth #17, the presence of a palatal abscess without an evident fluctuation point, negative pulp vitality testing (Roeko; Coltene Whaledent, Langenau, Germany), and pain on percussion and palpation. Periodontal exams confirmed normal probing depth all around the tooth and normal physiologic mobility. Radiographic analysis showed a large coronal restoration, preserved lamina dura, slight thickening of the periodontal ligament space, and incomplete root development (Figure 1A–F). The diagnosis of periapical abscess was established for tooth #17. Considering the immaturity of the tooth, pulp revascularization was the recommended treatment approach. The treatment procedures, potential risks, and expected benefits were explained to both the guardian and the patient in language appropriate to the patient’s age and level of understanding.

Due to extensive loss of tooth structure, a crown lengthening procedure was performed to reestablish the biological width, given the invasion of the supracrestal attachment. A trans‐surgical elevation of the gingival margin was performed with composite resin. Prior to the procedure, intraoral antisepsis was carried out with 0.12% chlorhexidine digluconate (Riohex Guard, Rioquímica, São Paulo, SP, Brazil), and extraoral antisepsis with 2% chlorhexidine digluconate (Riohex, Rioquímica, São Paulo, SP, Brazil).

Periodontal probing was performed using a North Carolina Probe (Hu‐Friedy, Leimen, Germany) to measure the distance between the alveolar crest and the future gingival margin, determining the amount of cervical tissue to be excised. Local anesthesia was achieved via posterior superior alveolar nerve block and supplemental infiltrations in the papillae and marginal regions, using 2% lidocaine with 1:100,000 epinephrine (DFL Indústria e Comércio, Rio de Janeiro, RJ, Brazil). An internal bevel incision was made with a #12 scalpel blade to redefine the gingival margin, followed by an intrasulcular incision. The cervical collar was removed using a Gracey 7–8 curette (Hu‐Friedy, Leimen, Germany), and a full‐thickness flap was raised using a Molt 2/4 periosteal elevator (Hu‐Friedy, Leimen, Germany). Osseous recontouring was performed using a Micro Ochsenbein chisel #1 (Hu‐Friedy, Leimen, Germany) to re‐establish a biological width of 3 mm. The extensive restorative material was removed, and a gingival margin was elevated with Filtek One Bulk Fill composite resin in A2 shade (3 M, São Paulo, SP, Brazil). The procedure was completed with horizontal suspensory sutures using 6–0 nylon thread (Shalon, São Luís de Montes Belos, GO, Brazil) (Figure 1G–O). Postoperative instructions regarding oral hygiene and care were provided, along with a prescription for pain and inflammation control: dipyrone 500 mg every 6 h for 3 days, if needed; nimesulide 100 mg every 12 h for 3 days; and 0.12% chlorhexidine mouthwash twice daily for 7 days.

**Figure 2:**
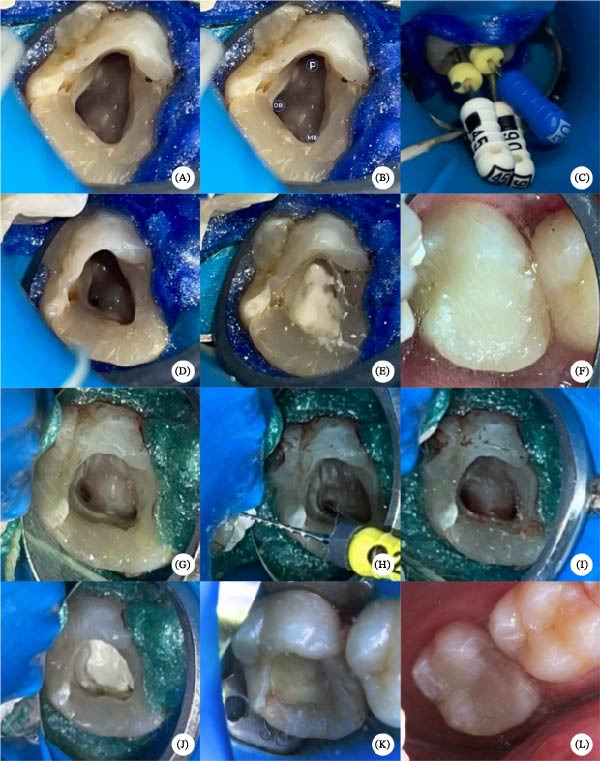
Endodontic treatment with regenerative protocol (pulp revascularization). (A–C) Access cavity preparation under rubber dam isolation and identification of the root canals. (D–F) Root canal disinfection procedures and placement of intracanal medication with provisional sealing. (G–I) Removal of intracanal medication, canal drying, and induction of apical bleeding to promote formation of an intracanal blood clot scaffold. (J–L) Placement of a bioceramic material over the blood clot, followed by coronal sealing and definitive composite resin restoration.

Endodontic access was then achieved with a #1014 round diamond bur (KG, São Paulo, SP, Brazil), and chamber refinement was performed using an Endo‐Z bur (Angelus, Paraná, PR, Brazil), enabling identification of three root canals: mesiobuccal, distobuccal, and palatal. Absolute isolation was established using a rubber dam and a supplementary gingival barrier (Biodam, Biodinâmica, Paraná, PR, Brazil) (Figure 2A–C). Canal debridement were performed using K‐files (Dentsply Maillefer, Ballaigues, Switzerland). Irrigation of the canal was performed using 2.5% sodium hypochlorite (NaOCl; Asfer, São Paulo, SP, Brazil), followed by a final flush with 17% EDTA (Biodinâmica, Itaporã, PR, Brazil) and neutralization with saline solution. Intracanal medication consisted of calcium hydroxide paste (Ultracal, Ultradent, São Paulo, SP, Brazil), maintained for 30 days under provisional sealing using Bulk Fill Flow composite resin (Solventum, São Paulo, SP, Brazil) (Figure 2D–F).

After 30 days, the patient was asymptomatic, the tooth was not sensitive to palpation and percussion, and the swelling was resolved. Under local anesthesia and rubber dam isolation, the provisional restoration and calcium hydroxide paste were removed, and the canals were dried with sterile paper points (Dentsply Maillefer, Ballaigues, Switzerland). Apical bleeding was induced using small‐size #20 Hedstroem file (Dentsply Maillefer, Ballaigues, Switzerland) to facilitate blood clot formation within the root canal space. After 10 min, a silicate‐based material (Biodentine; Septodont, Saint‐Maur‐des‐Fossés, France) was mixed according to the manufacturer’s instructions, and carefully placed over the blood clot. The access cavity was then filled with glass ionomer cement (Bioglass; Biodinâmica, São Paulo, SP, Brazil), followed by definitive restoration using Filtek Z350 composite resin in shades EA2 and DA3 (Solventum, São Paulo, Brazil) (Figure 2G–L).

**Figure 3:**
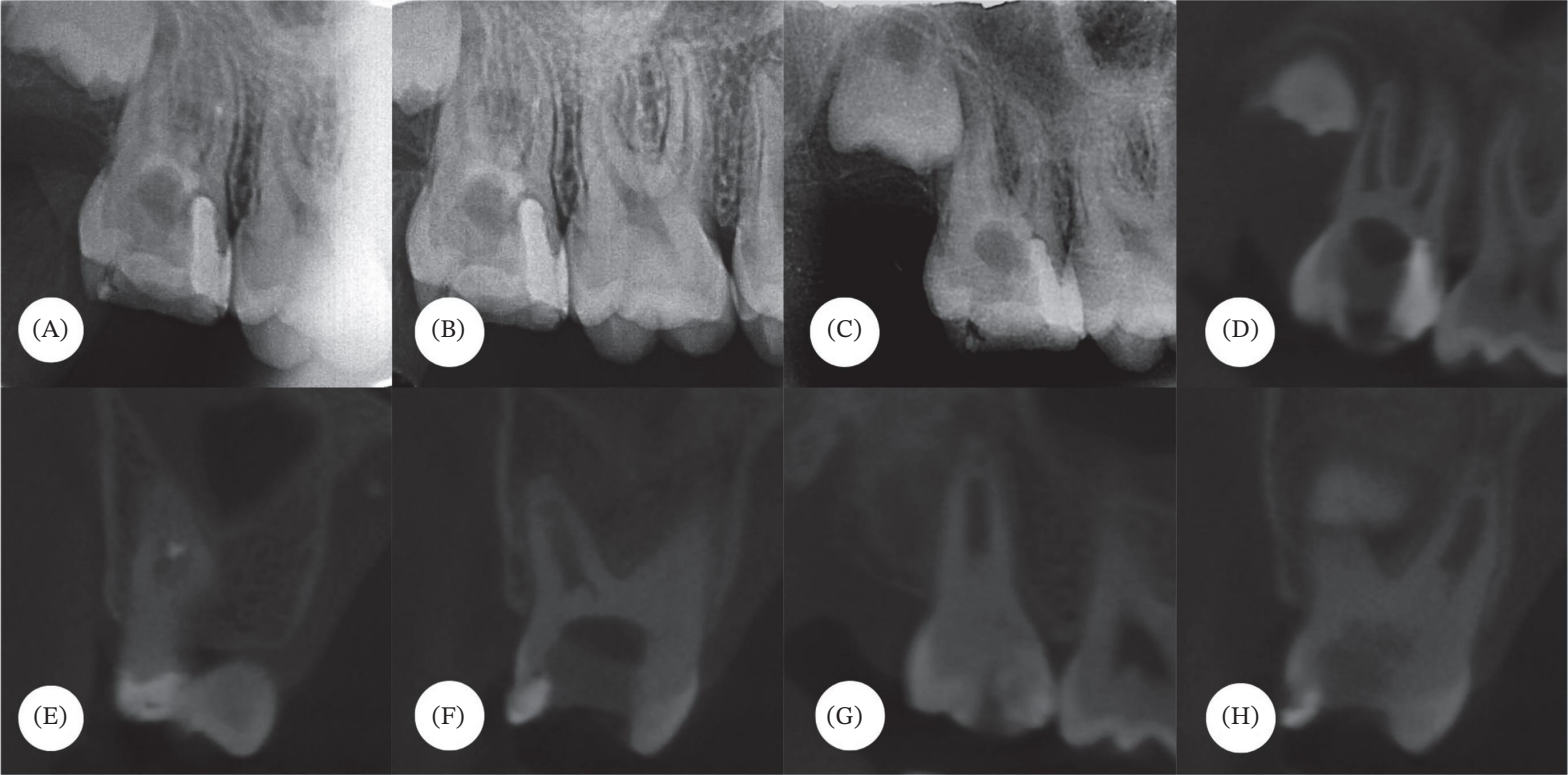
Radiographic and tomographic follow‐up after regenerative endodontic treatment. Follow‐up examinations. (A) Immediate postoperative periapical radiograph. (B) Radiographic follow‐up after 3 months. (C) Radiographic follow‐up after 8 months. (D–H) Cone‐beam computed tomography (CBCT) images obtained after 2 years demonstrating apical closure and evidence of continued root development.

The patient was recalled at 3, 8, and 24 months after treatment. Tooth #17 remained asymptomatic and functional, with no recurrence of swelling reported. Radiographic and tomographic evaluations confirmed the absence of infection and inflammation, along with evidence of continued root development (Figure 3A–H).

## 3. Discussion

Dental caries is a highly prevalent condition among children and adolescents, impacting not only oral health but also overall quality of life [11]. Extensive carious lesions require prompt clinical management due to the risk of severe pain and pulp involvement, which can progress to periapical infections and as seen in the present case [4]. In developing permanent teeth, preserving pulp vitality is essential for continued root maturation and apical closure [1, 2]. This consideration is particularly critical in teeth with incomplete root formation, as interruption of this process may result in long‐term structural and functional deficits [1, 2].

Irreversible pulp damage leads to necrosis, halting root development and increasing the risk of complications such as periapical abscess [4]. These lesions are marked by the presence of pathogenic microorganisms and tissue degradation, which can result in facial swelling due to accumulation of purulent exudate [4, 10]. Patients may present a firm, nonfluctuant swelling, indicating active inflammation and disease advancement. As the condition progresses, timely and more complex interventions become necessary to avoid further clinical complications [4].

Accessing and managing immature teeth with open apices is technically challenging and demands a specialized, meticulous approach. The primary objective is to establish a biologically favorable environment that supports cellular proliferation and tissue regeneration, while simultaneously achieving effective disinfection of the root canal system to eliminate microbial interference [12]. In the present case, pulp revascularization was the most appropriate treatment option, particularly when compared to traditional apexification [8]. Although apexification has been widely used for immature teeth with necrotic pulps, it primarily induces the formation of a calcified apical barrier without promoting further root development [1, 2]. Consequently, roots often remain thin and structurally vulnerable, compromising long‐term tooth survival [13]. Revascularization has been proposed as a biologically based treatment option capable of promoting continued root development and apical closure in immature teeth. In teeth with open apices, this approach not only resolves infection but also facilitates apical closure, and reinforcement of the root structure, ultimately improving the tooth’s long‐term prognosis and biomechanical resistance [1, 2, 5–7, 13].

REPs in immature teeth may present several technical challenges for clinicians due to the anatomical characteristics of teeth with open apices and wide root canals [1, 2]. Effective disinfection of the root canal system is particularly critical in these cases, as residual microorganisms may compromise the regenerative process and interfere with tissue healing [4, 12]. Another important step is the induction and stabilization of an intracanal blood clot, which serves as a biological scaffold that supports cell migration, proliferation, and tissue regeneration [1, 9]. In addition, achieving a reliable coronal seal is essential to prevent bacterial reinfection and to maintain a favorable environment for continued root development [2, 14]. Previous studies have emphasized that strict adherence to the clinical protocol and appropriate case selection are key factors influencing the success of regenerative endodontic therapy [1, 12].

In this case, the revascularization protocol was carefully executed [15]. The root canal system, including the mesiobuccal, distobuccal, and palatal canals, was accessed and instrumented. Irrigation was performed with 2.5% sodium hypochlorite, applied 4 mm short of the working length to ensure disinfection while minimizing the risk of extrusion beyond the apex. A 30‐day intracanal medication with calcium hydroxide was employed to reduce bacterial load and promote tissue remineralization. Apical bleeding was induced with a size #20 Hedstroem file to initiate the formation of a stable blood clot within the canal space, serving as a biological scaffold essential for cell migration and tissue regeneration. This step was critical to the success of the regenerative procedure [1, 2, 5–7]. Biodentine, a tricalcium silicate‐based bioceramic material, was used to seal the coronal portion of the canal. Recognized for its excellent biocompatibility, ability to stimulate mineralization, and reliable sealing capacity, Biodentine contributes to favorable healing outcomes [14]. A glass ionomer base was placed, followed by a composite resin restoration to reestablish function and esthetics.

Cone‐beam computed tomography (CBCT) played a crucial role during the 2‐year follow‐up by providing a detailed assessment of root maturation and apical closure. CBCT enabled precise visualization of the three root canals and confirmed ongoing root development, even in the presence of initial periapical pathology and incomplete apices [16]. Previous studies have also reported successful outcomes of REPs based primarily on radiographic and clinical observations [1, 2, 6, 7]. Unfortunately, quantitative measurements of root length, dentinal wall thickness, or apical diameter were not performed. Future investigations incorporating standardized morphometric analyses may provide more objective evidence regarding structural changes following REPs.

Although the present case demonstrated favorable clinical and radiographic outcomes after a 2‐year follow‐up period, longer observation intervals are recommended when evaluating REPs. Continued root maturation, dentinal wall thickening, and apical closure may progress for several years following treatment [1, 2, 9]. Therefore, long‐term follow‐up remains essential to confirm the stability and functional prognosis of teeth treated with regenerative protocols.

## 4. Conclusion

Within the limitations inherent to a single case report, the present case demonstrates that regenerative endodontic therapy may represent a viable treatment option for immature permanent molars with necrotic pulp and periapical infection. In this case, the regenerative protocol allowed infection control, continued root development, and apical closure after 2‐year follow‐up. Nevertheless, further clinical studies with larger samples are required to confirm the predictability of this approach.

## Funding

No funding was received for this manuscript.

## Ethics Statement

This study followed the ethical principles of the Declaration of Helsinki and ensured patient confidentiality and welfare.

## Consent

Written informed consent for treatment and publication of the clinical data and images was obtained from the patient’s legal guardian. The procedures, potential risks, and expected benefits were explained to both the guardian and the patient in language appropriate to the patient’s age and level of understanding. The patient also provided assent to the proposed treatment.

## Conflicts of Interest

The authors declare no conflicts of interest.

## Data Availability

The data that support the findings of this study are available from the corresponding author upon reasonable request.
